# Sodium reduction strategies through use of meat extenders (white button mushrooms vs. textured soy) in beef patties

**DOI:** 10.1002/fsn3.824

**Published:** 2019-01-21

**Authors:** Kristin M. Wong, Maria G. Corradini, Wesley Autio, Amanda J. Kinchla

**Affiliations:** ^1^ Department of Food Science University of Massachusetts Amherst Massachusetts; ^2^ Stockbridge School ofAgriculture University of Massachusetts Amherst Massachusetts

**Keywords:** beef patties, meat extension, mushroom, physical properties, sensory, sodium reduction

## Abstract

Lowering the sodium content in meat products, particularly in beef patties, can be challenging because sodium plays many functional roles in these products. Meat extenders can contribute to lower sodium content by imparting complementary flavors while reducing caloric and sodium content. A systematic comparison of two meat extenders, namely mushrooms and textured soy (TSP) in terms of physical and sensory characteristics, is presented herein. The physical properties of the samples suggested that the use of mushroom and TSP extender would perform statistically similar to an all‐meat control depending on the level of substitution. Hedonic sensory analysis showed meat extension using mushrooms yielded liking scores more similar to the all‐meat formulations than TSP in reduced sodium applications. The results of this research suggest that mushrooms have the potential to be successfully incorporated into reduced sodium meat products to provide a healthier product.

## 1 INTRODUCTION

Excess consumption of sodium can increase blood pressure and the risk of cardiovascular disease and stroke. Cardiovascular disease is the leading cause of death in the United States while stroke is the fifth leading cause (Centers for Disease Control and Prevention, 2016). The U.S. Department of Agriculture (2015) Dietary Guidelines for Americans recommends shifting to low sodium eating patterns to help reduce the risk of these detrimental diseases. The average American consumes 3,400 milligrams of sodium, which is almost 1.5 times higher than the recommended upper limit of 2,300 milligrams. The largest source of sodium in the American diet comes from the beef and sandwich food category at 21% (U.S. Department of Agriculture and U.S. Department of Health and Human Services, 2015). Beefs and sandwiches are a major target for sodium reduction due to their large contribution to the overall sodium intake in the American diet.

Although various strategies have been developed to reduce sodium in the meat industry, the proposed options come with different advantages and disadvantages. Lowering the sodium content in meat products can be a challenge because sodium, in the form of sodium chloride, or salt, plays many functional roles. Salt can enhance flavor, improve texture through water binding, and extend shelf life by limiting microbial growth in meat products (Ruusunen & Puolanne, 2005; Desmond, 2006; Terrell, 1983; Madril & Sofos, 1985). When formulating reduced sodium products, developers must adjust the composition and incorporate ingredients to compensate for the loss of functionality due to salt removal. According to Desmond (2006), three major categories are used in the meat industry to reduce sodium content: salt substitutes, flavor enhancers, and salt with modified structures. Among the salt substitutes, potassium chloride is the most commonly used in reduced sodium products; however, it cannot replace more than 50% of the sodium chloride in a product without imparting bitterness to the final product (Desmond, 2006). Phosphates also lower the requirement of added sodium in meat products while improving water binding and cook yield (Barbut, Maure, & Lindsay, 1988; Puolanne & Terrell, 1983; Trout & Schmid, 1984; Ruusunen, Niemisto, & Puolanne, 2002; Ruusunen et al., 2005). However, food manufacturers are currently inclined to remove phosphates from their products to cater to consumers’ demands for cleaner labels (Edwards, 2013; Bobe & Michel, 2011; Hoogenkamp, 2012; Cheung et al., 2016). Taste enhancers help to compensate for lower sodium content by activating taste receptors in the mouth (Brandsma, 2006). Yeast extract, lactates, monosodium glutamate, and nucleotides can be used in combination with salt substitutes, particularly potassium chloride, to help mask bitter or metallic flavors (Grummer, Bobowski, Karalus, Vickers, & Schoenfuss, 2013; Campagnol, Alves dos Santos, Wagner, Terra, & Pollonio, 2011; Ruusunen, Simolin, & Puolanne, 2001; Desmond, 2006). However, according to Desmond (2006), these ingredients can impart their own flavor, which can be undesirable in certain products and limit their application. Finally, solid salt's crystal size and shape can be manipulated to alter the perception of saltiness in meat products so less can be used (Angus et al., 2005; Desmond, 2006). Salt flakes can improve cook yield through water binding due to its higher solubility than granular salt, which can be beneficial to low moisture meat applications (Campbell, 1979; Lutz, 2005). Although some of these sodium reduction strategies have been adopted by the meat industry, to improve health, new strategies must be developed to lower sodium consumption in meat products without compromising on quality and taste. The use of meat extenders poses a dual opportunity by reducing caloric content while also imparting flavors that can complement and enhance saltiness perception.

The use of mushrooms as meat extenders to reduce caloric content has gained popularity in recent years. The flavor‐enhancing characteristics of mushrooms have the potential to become a strategy to reduce sodium in meat products. Myrdal Miller et al. (2014) successfully showed that mushrooms can be used to mitigate flavor loss in sodium‐reduced products, however, with limited application to beef taco filling’. Therefore, there is an opportunity to expand the investigation of using mushrooms in other applications. Previous work on the incorporation of mushrooms into beef and chicken patties resulted in improved physical qualities while deemed acceptable to untrained panelists during sensory evaluation (Wan Rosli, Solihah, Aishah, Nik Fakurudin, & Mohsin, 2011; Wan Rosli and Solihah, 2012, 2014). Although this work targeted the beef food category, it did not study the efficacy of mushroom incorporation as a sodium reduction strategy.

Soy is a commonly used meat extender that can improve the quality of meat‐based products. Soy can be added to meat products in a variety of different forms with varying protein concentrations: soy flour (50% protein), soy concentrate (70% protein), or soy protein isolate (90% protein) (USDA National Nutrient Database, 2016). Since soy is a protein‐based meat extender, it has the ability to bind with water and fat to produce products with higher moisture content and yield (Brewer, 2012). Studies have shown that soy substitution up to 20% in beef products can decrease cooking loss and evaporative loss (Dignam, Tseng, & Smith‐Nury, 1979; Kilic, Kankaya, Ekici, & Orhan, 2010). However, the sensory attributes of the final product can be compromised when soy is added at high concentrations in some applications. Akesowan (2010) reported decreases in flavor liking and increased bean flavor when soy protein isolate was added to pork patties. Danowska‐Oziewicz (2014) observed that sensory panelists noted decreased meaty flavor at 2% soy protein isolate substitution and increased bean flavor at 5% substitution low fat pork patties.

The objective of this study was to conduct a systematic study of the effects of the addition of white button mushrooms (*Agaricus bisporus*) and a commonly used meat extender on the physical and sensory attributes of beef patties. We investigated the effect of mushroom's flavor characteristics as a sodium reduction strategy in comparison with textured soy in beef patties and an all‐meat formulation.

## 2 MATERIALS AND METHODS

### 2.1 Suppliers and ingredient preparation

Arnold's Meats (Chicopee, MA) supplied 80/20 blend ground beef. The ground beef was used without any further preparation to form the all‐meat and substituted formulations. White button mushrooms were selected due to their distributional access and affordability. Individually quick frozen (IQF), 9.5 mm diced, white button mushrooms (immature *Agaricus bisporus*) were also supplied by Arnold's Meats. The IQF mushrooms were placed into a food processor (Cuisinart, East Windsor, NJ) for 6 one‐second pulses to obtain small particles (length = 1 to 5 mm). This protocol yielded 95% to 99% of particles in the desired size range based on AOAC Method 973.03, which established protocol to determine particle size ranges. Texture soy protein concentrate (TSP) was sourced from Solae (Response 4320, St. Louis, MO). The TSP was caramel colored and ranged in size from 2 to 6 mm. Before beef patty formulation, the TSP was hydrated at a 1:1 volume ratio (1.0:2.4 TSP to water ratio by gram weight) with hot water in a stand mixer (KitchenAid, Benton Harbor, MI) with a paddle attachment on low speed for 5 min. The hydrated TSP (h‐TSP) particle size did not change after reconstitution. The particle size of both meat extenders added to the patties was comparable (1–5 mm vs. 2–6 mm). Salt was purchased from a local supplier.

### 2.2 Composition and physical properties of patties

#### 2.2.1 Formulation, shaping, and cooking

The composition of beef patties with varying ratios of 80/20 ground beef and meat extender, either mushroom or h‐TSP, is summarized in Table 1. Appropriate amounts of ground beef and meat extender were placed in a stand mixer (KitchenAid, Benton Harbor, MI) with a dough hook attachment and mixed on a low speed for 5 min. Once homogeneous, the formulation was divided into 56.7 g portions and shaped using a mini beef press (Norpro, Everett, WA). Patties were shaped to a uniform size with a 65 mm diameter and 17 mm thickness. Patties were then placed in a 305 mm diameter aluminum frying pan (Pedrini, Lifetime Brands, Garden City, NY) on an electric range (Kenmore 94173, Kenmore, Chicago, IL). Patties were cooked on one side at medium heat for 3 min, flipped, and cooked for an additional 3 min until the internal temperature reached 74°C. Internal temperature was taken by inserting a temperature probe (Thermo Fischer Scientific, Waltham, MA) into the center of the patty through the side.

**Table 1:** Ground beef blend and meat extender (mushroom or h‐TSP) ratios by weight for physical characterization tests

Formulation	Ground Beef (% w/w)	Meat Extender Type	Meat Extender (% w/w)
1 (Control)	100	–	–
2	90	Mushroom	10
3	80	Mushroom	20
4	70	Mushroom	30
5	60	Mushroom	40
6	50	Mushroom	50
7	90	Textured Soy	10
8	80	Textured Soy	20
9	70	Textured Soy	30
10	60	Textured Soy	40
11	50	Textured Soy	50

*Note*. Formulations were prepared with a maintained salt (NaCl) concentration of 1.5%.

#### 2.2.2 Cook yield

Cook yield was determined by measuring the weight of each patty before and after cooking. The values were inserted in Eq. (1) (Wan Rosli et al., 2011). The results are reported as percentage:

(1) $$\text{Cook yield}\left(\%\right)=\frac{\text{Cooked weight}}{\text{Pre‐cooked weight}}*100$$

#### 2.2.3 Moisture content and retention

Moisture content was determined using AOAC Method 950.46 A (Official Methods of Analysis of AOAC International 2012a). 2 ± 0.01 grams of patty was placed in a 57.2 mm diameter aluminum, weighing dish (Scientific Equipment of Houston, Navasota, TX) and introduced in a vacuum oven (Lab‐Lane Instruments, Melrose Park, IL) connected to a rotary vacuum pump (FJC, Mooresville, NC). Oven temperature was 100°C and pressure was 100 mm Hg. Drying continued until the weight of the samples was constant. Moisture content is reported as percent moisture (%). Moisture retention was calculated using Eq. (2), and results are reported percent moisture retained (El‐Magoli, Laroia, & Hansen, 1996):

(2) $$\text{Moisture retention}\;\left(\%\right)=\frac{\%\text{Yield}*\%\text{Moisture in cooked patty}}{100}*100$$

#### 2.2.4 Color analysis

Color of samples was measured using a colorimeter (ColorFlex EZ™, Hunter Lab, Reston, VA) on the L*a*b* scale. Each patty was placed on a plastic petri dish (Thermo Fischer Scientific, Waltham, MA) and covered with a black, metal cup to provide a consistent, black background. The instrument was calibrated with a white Illuminant D65 10° Observer ASTM E308: X: 79.59, Y: 84.44, and Z: 87.25 standard. Results are reported without units.

#### 2.2.5 Mechanical properties

A stress–relaxation test was performed to assess the mechanical properties of the patties (Peleg, 2006; Suwonsichon & Peleg, 1999; Ak & Gunasekaran, 1995). Each patty was compressed using a Universal Testing Machine (UTM) (Model 5542, Instron, Norwood, MA) equipped with a circular metal plate (50 mm diameter). The crosshead displacement was set at a speed of 5 mm min^−1^. Patties were compressed to 50% of their initial height (50% deformation). After this height was reached, the specimen was allowed to relax for 120 s before the crosshead was withdrawn. Two metrics were recorded for evaluation: apparent stress at 50% deformation and apparent stress at the end of a 2‐min relaxation period, dubbed residual apparent stress. Results are reported in kPa.

#### 2.2.6 Sodium content analysis

Sodium content analysis was executed using an ion selective electrode (Thermo Fischer Scientific, Waltham, MA) based on the AOAC Method 976.25 (Official Methods of Analysis of AOAC International 2012b). Results are reported as milligrams of sodium per gram of sample.

#### 2.2.7 Fat content and retention

Fat content of the samples was analyzed by extraction with diethyl ether using a Soxhlet apparatus with Allihn condenser (Thermo Fischer Scientific) based on AOAC Method 960.39 (Official Methods of Analysis of AOAC International 2012c). Before extraction, the specimen was placed in a −40°C freezer (Environmental Equipment Company, Cincinnati, OH) for at least 24 hours. Thoroughly frozen samples were then freeze‐dried (Virtis Consol 12LL, The Virtis Company Inc., Gardiner, NY) and ground to a fine powder using a grinder (Krups F203 Grinder, Krups, Groupe SEB, Ecully, France). Powder samples were then used for the analysis. Fat content is reported as percent fat. Fat retention was calculated using Eq. (3), and results are reported as percent fat retained (El‐Magoli et al., 1996):

(3) $$\text{Fat Retention}\left(\%\right)=\frac{\text{Cooked patty weight}*\%\text{Fat in cooked patty}}{\text{Raw patty weight}*\%\text{Fat in raw patty}}*100$$

### 2.3 Sensory test

Two hedonic sensory tests were conducted to measure the consumer acceptability of the different beef patty formulations (Table 2). The initial sensory test fielded included a series of meat extender concentrations (one all‐beef control and six meat extender products) to determine which meat extender formulas (1 mushroom, 1 h‐TSP) would most resemble the sensory attributes of an all‐beef product. Product formulations were prepared at a “full sodium” level set at 1.5% salt by weight which as determined based on the salt range (0.8%–2.0%) used in other published work on beef patties (Tobin, O'Sullivan, Hamill, & Kerry, 2012; Wan Rosli and Solihah, 2012, 2014, 2014; Angor & Al‐Abdullah, 2010; Barbosa et al., 2015). The “reduced sodium” level used in the Hedonic Test #2 product formulations was set at 1.1% by weight based on the calculations to meet the regulatory requirements for a “reduced sodium” claim (i.e., to have “25% less sodium per RACC than an appropriate reference food”) (U.S. Food and Drug Administration, 2013). Using the results from the first sensory test (Hedonic Test #1), an additional sensory trial (Hedonic Test #2) was conducted to measure consumer liking of reduced salt product formulations against a full salt, all‐beef control. Hedonic testing was selected to evaluate the degree of acceptability of the meat extender products (mushroom and h‐TSP) against a traditional 100% beef.

**Table 2:** Ground beef (80/20 blend) and meat extender (mushroom or textured soy) formulations by weight for each hedonic sensory study

	Formulation	Ground Beef (% Weight)	Meat Extender Type	Meat Extender (% Weight)	Salt (% Weight)
Meat Extender Concentration Optimization (Hedonic Sensory Test #1)	1 (Control)	98.5	–	–	1.5
	2	88.5	Mushroom	10	1.5
	3	78.5	Mushroom	20	1.5
	4	68.5	Mushroom	30	1.5
	5	88.5	Textured Soy	10	1.5
	6	78.5	Textured Soy	20	1.5
	7	68.5	Textured Soy	30	1.5
Reduced Sodium Patties With Meat Extenders (Hedonic Sensory Test #2)	1 (Control)	98.5	–	–	1.5
	2	98.9	–	–	1.1
	3	78.9	Mushroom	20	1.1
	4	79.9	Textured Soy	20	1.1

#### 2.3.1 Sample preparation for hedonic sensory test

Beef and meat extender formulations were mixed following the same procedures as stated in Section 2.2.1. Once homogeneous, each formulation was divided into 114 g portions and shaped by hand to a uniform shape (approximately 120 mm diameter and 15 mm thickness). Patties were then stored at −18°C in between sheets of wax paper in plastic bags until further use.

For convenience and appropriate representation of commercially available patties, the sample size was increased for sensory analysis. The cooking time was adjusted accordingly to provide the equivalent heat treatment and additional verification of the physical samples was confirmed prior to fielding the sensory analysis. Frozen patties were then placed in a 305 mm diameter aluminum frying pan (Pedrini, Lifetime Brands, Garden City, NY) on an electric range (Kenmore 94173, Kenmore, Chicago, IL). Patties were cooked on one side at medium heat for 7 min, flipped, and cooked for an additional 7 min until the internal temperature reached 74°C. Internal temperature was taken as described in section 2.2.1. Cooked patties were then quartered and placed onto a grate in a slow cooker (Bella 13972, Bella Housewares, Cape Town, South Africa) filled with 20 mm of water to maintain patty temperature at 65°C–72°C and moisture content at 60% to 65% w/w (United States Department of Agriculture, 2007) until they were served to the panelists.

#### 2.3.2 Hedonic sensory testing

Sensory evaluations followed the American Society for Testing and Materials (ASTM) Sensory Evaluation Standards (ASTM Method E2943‐15) and had approval from the University of Massachusetts Institutional Review Board (IRB) for the Protection of Human Subjects prior to fielding these experiments. Hedonic acceptability tests were fielded at the UMass Food Science Chenoweth Laboratory following a sequential, monadic test method. Prior to participating in testing, subjects were screened to confirm that they were consumers that eat beef patties. Test subjects were seated at isolation stations to provide a consistent test environment and reduce bias from the presence of other participants. Untrained consumer panelists from the UMass campus were recruited for two hedonic tests [*N* = 55 (Hedonic Test #1) and *N* = 56, respectively (Hedonic Test #2)]. Sensory testing was implemented using Sensory Information Management System (SIMS) 2000 software V6.0 (Sensory Computer Systems LLC, Berkeley Heights, NJ).

Each test subject was independently served a quarter of a patty sample of the control and three variant formulations on 152 mm white paper plates at 66°C to 71°C and a cup of water. Panelists were served water and nonsalted saltine crackers between sample evaluations to cleanse their palates. Test subjects used a ballot to evaluate each of the samples using a 9‐point hedonic scale (1 = extremely dislike, 5 = neutral, and 9 = extremely like) (Peryam & Pilgrim, 1957). The attributes evaluated included overall liking, aroma, color, flavor, juiciness, saltiness, and texture modeled after other published meat‐based sensory tests (Tobin et al., 2012; Barbosa et al., 2015; Saricoban, Yilmaz, & Karakaya, 2009).

##### Hedonic sensory test to assess consumer liking of beef patties with extenders (Hedonic Test #1)

The initial sensory test (Hedonic Test #1) included a series of beef patty formulations (seven products) with varying ratios of beef and h‐TSP or mushroom to first measure consumers’ acceptance of meat extenders using a block design using the SIMS 2000 software described above. Within each of the test, subjects randomly evaluating four of the seven tested formulations to reduce palate fatigue.

##### Hedonic sensory test to measure consumer liking of meat extenders with reduced salt (Hedonic Test #2)

The results from the Hedonic Test #1 sensory trial helped to guide product formulations selected that used h‐TSP and mushroom to perform a second sensory test (Hedonic Test #2) to then measure consumers’ response to full salt and reduced salt product formulations compared to a full salt, all‐beef beef patty. Using the same ballot and sampling protocols described above, panelists randomly evaluated all four beef patties of the tested formulations.

### 2.4 Statistical analysis

Three replications with two measurements were taken on each patty formulation for each of the physical properties tested. The order of analysis for each variant formulation was randomized to reduce order bias. The objective of the physical analysis was to compare each variable against the control to identify which variable was most similar control; therefore, data from the physical analyses were evaluated using analysis of variance (ANOVA) and Dunnett's Test with SAS 9.4 Windows version 6.1.7601 (SAS Institute Inc., Cary, NC). The ANOVA was selected to identify differences among the variant formulations and the all‐meat control for each physical test; however, due to the limitations of the ANOVA, it could not determine whether or how many variant formulations significantly differed from the all‐meat control. When significant differences were found using the ANOVA, the Dunnett's Test was conducted to directly compare each variant formulation to the all‐meat control and identify specific, significant differences. The ANOVA main effect for the physical attributes focusing on meat extender type and concentration was “meat extender type,” “meat extender concentration,” and “replication”. The all‐meat control (0% meat extender) was included in this analysis. Each variant formulation and the all‐meat control were analyzed as “treatments” for the Dunnett's Test.

Data from the first hedonic sensory study were also evaluated using a two‐way ANOVA test to identify differences in liking scores among the all‐meat control and the product formulations (studying the interactions of the extender type [mushroom and soy] and ration of extender [10, 20 and 30%]). When statistical differences were observed, a Tukey's honestly significant difference (HSD) test at 95% confidence was conducted to further investigate the formula interactions. Results for the second hedonic sensory study were analyzed using an ANOVA test to identify differences in liking scores among the variant formulations (all‐meat with reduced salt, 20% mushroom with reduced salt, 20% soy with reduced salt) and the all‐meat full salt control. Further data analysis with Duncan's New Multiple Range Test was conducted to compare the liking scores of the variant formulations not only to the all‐meat control but also to each other. This test was selected to detect differences in liking from the all‐meat control as well as identifying any thresholds in liking across a range of meat extender type and concentration.

## 3 RESULTS AND DISCUSSION

### 3.1 Effect of meat extender type and concentration on beef patty physical characteristics

Physical attributes of all samples were determined to assess the effect of meat extender type and concentration on beef patties. The purpose of these tests was to create a formulation using meat extenders as similar as possible to the all‐meat control. IQF white button mushrooms and h‐TSP were separately added to beef patties as indicated in Table 1. Statistical analysis was conducted for the results of the physical attributes to determine differences among formulations varying in meat extender type and concentration. The ANOVA *p*‐values are summarized in Table 3. Meat extender type had a significant effect on six out of nine physical attributes: moisture content, moisture retention, cook yield, yellow color (b*), and texture (apparent stress at 50% deformation and residual stress). Meat extender concentration also influenced six out of nine attributes: moisture content, yellow color (b*), texture (apparent stress at 50% deformation and residual stress), sodium content, and fat content (Table 3). Type of meat extender only influenced three out of nine.

**Table 3:** Statistical differences in physical attributes among burger patty formulations using varied meat extender types and concentrations evaluated by meat extender type, concentration, and type by concentration

Source of Variation	Moisture Content	Moisture Retention	Cook Yield	L*	a*	b*	Apparent Stress at 50% Deformation	Residual Stress	Sodium Content	Fat Content	Fat Retention
Meat Extender Type	**0.0004** *	**0.0060** *	**0.0009** *	0.1677	0.2362	**0.0046** *	**0.0001** *	**0.0003** *	0.0939	0.0512	0.1771
Meat Extender Concentration	**<0.0001** *	0.0631	0.3696	0.2504	0.0712	**0.0038** *	**0.0070** *	**0.0039** *	**0.0012** *	**0.0012** *	0.0971
Type* Concentration	0.8330	**0.0134** *	**0.0007** *	0.5610	**0.0047** *	–	0.7330	‐	‐	0.0501	0.4616

*Note*. ANOVA significance at *p ≤ *0.05 is shown with an * in bold.

The results of each physical attribute for different formulations are further discussed in the following sections.

#### 3.1.1 Moisture content and retention

**Figure 1:**
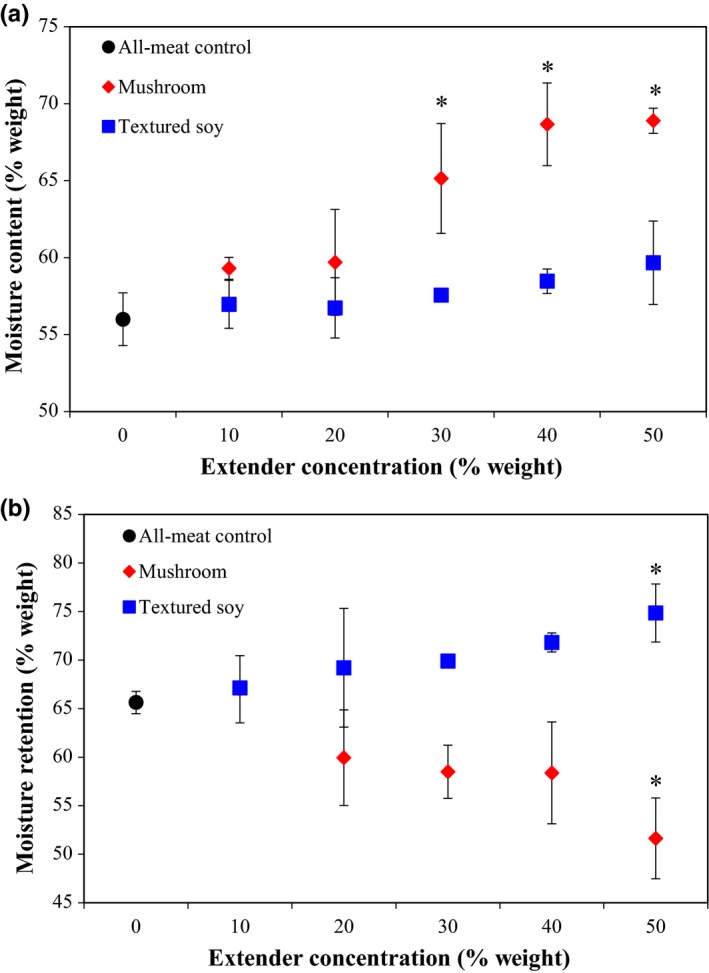
Effect of meat extender type (mushroom or textured soy) and concentration on (a) moisture content and (b) moisture retention. *Note*: The black circle corresponds to the all‐meat control (0% extender). Error bars presented are the calculated standard deviations within each treatment. Data points with an *indicate a significant difference from the control (Dunnett, *p *=* *0.05)

As presented in Table 3, both meat extender type and concentration had a statistically significant effect on moisture content. The Dunnett's Test showed that mushroom concentration at a substitution level equal or above 30% significantly increased moisture content when compared to the all‐meat control (see Figure 1a). This finding may be attributed to the higher moisture content found in mushrooms compared to ground beef, 90% and 60% w/w, respectively (U.S. Department of Agriculture, 2016). This observation is supported by the work of Wan Rosli and Solihah (2012) who found that 50% blanched oyster mushroom substitution in beef patties increased moisture content in the final product. It should be noted that moisture retention was not altered when chicken patties were substituted with 25% and 50% blanched oyster mushrooms (Wan Rosli et al., 2011). However, the chicken patty formulations did include 3% soy protein isolate with all formulations that may have increased the water holding capacity within these products (Wan Rosli et al., 2011). The addition of h‐TSP at all the levels tested did not have a significant effect on moisture content; that is, all substituted samples exhibited similar moisture contents as the all‐meat control (Figure 1a). The moisture content of the individual components of mushrooms, h‐TSP, and ground beef was 93.3%, 73.5%, and 62.84%, respectively. The relationship between TSP concentration and patty moisture content may be attributed to the hydrated TSP's moisture content being closer to ground beef than mushrooms. Previous studies found that soy substitution generally increased patty moisture content. Pork patties substituted with 10% soy protein isolate (SPI) and beef patties substituted with 2% TSP showed higher moisture content than their corresponding all‐meat controls (Danowska‐Oziewicz, 2014; Kassama, Ngadi, & Raghavan, 2003). Differences in patty moisture content have been attributed to several factors including meat type, soy type, soy rehydration ratio, cooking time, and cooking temperature.

A statistical difference in moisture retention among the different meat extenders was observed (Table 3). The Dunnett's Test revealed that beef patties with 50% mushroom substitution have statistically lower water retention, while 50% TSP beef patties have statistically higher water retention than the all‐meat control (Figure 1b). These findings suggest that the substitution of mushrooms or h‐TSP in beef patties at levels below 50% do not alter the overall moisture retention of the patty. At 50% substitution or above, beef‐mushroom and beef‐h‐TSP change the water holding capacity within the mixture due to the increased ratio of meat extender (mushroom or h‐TSP). Further research is necessary to determine the established relationship.

#### 3.1.2 Cook yield

**Figure 2:**
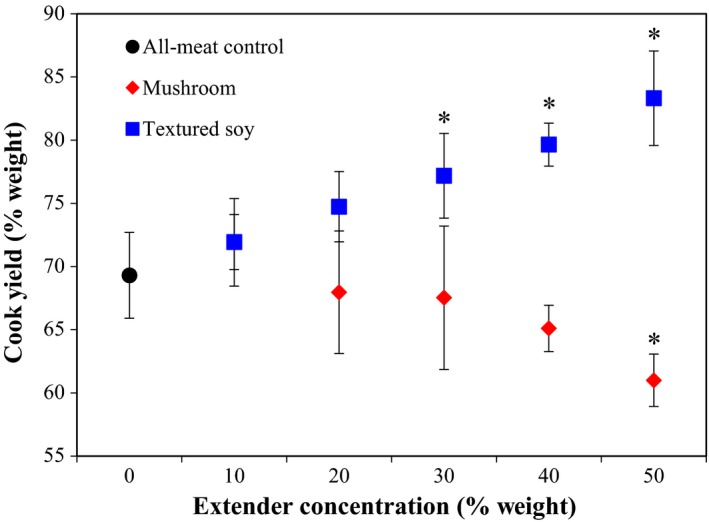
Effect of meat extender type (mushroom or textured soy) and concentration on cook yield. *Note*: The black circle corresponds to the all‐meat control (0% extender). Error bars presented are the calculated standard deviations within each treatment. Data points with an *indicate a significant difference from the control (Dunnett, *p *=* *0.05)

Significant differences in cook yield among the different meat extenders as well as an interaction between meat extender type and meat extender concentration were identified (Table 3). The Dunnett's Test analysis revealed a significantly lower yield in beef patties containing 50% mushroom than the all‐meat control. Wan Rosli and Solihah (2012) found similar reductions in cook yield for beef patties substituted with 25% and 50% blanched oyster mushrooms. However, chicken patties substituted with up 50% blanched oyster mushrooms did not display any changes in cook yield (Wan Rosli et al., 2011). Beef patties that included 20% or more TSP had significantly higher cook yields than the all‐meat control (Figure 2). The coefficient of variance (CV) of moisture content and moisture retention was 10% or lower, and these values are within the error of the methodology. The higher cooking yields from TSP are consistent with previous research which found increases in cook yield for 2% SPI in pork patties and 2% TSP in beef patties (Akesowan, 2010; Kassama, Ngadi, & Raghavan, 2003). Overall, TSP increased product yield, at concentrations as low as 20%, compared to mushrooms.

#### 3.1.3 Color analysis

**Figure 3:**
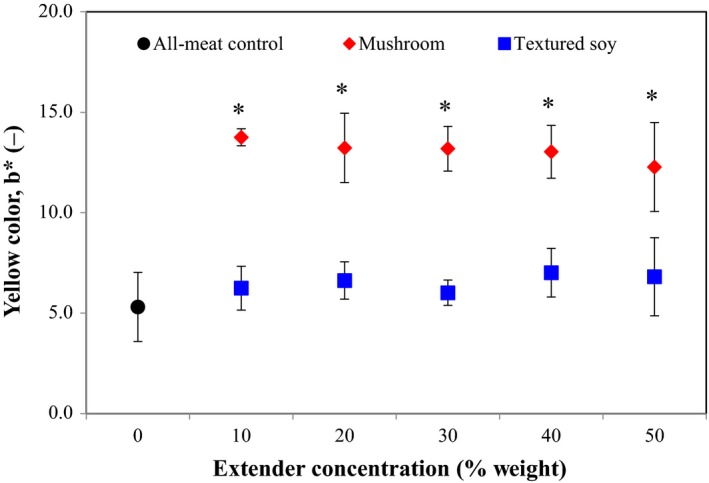
Effect of meat extender type (mushroom or textured soy) and concentration on yellow color (b*). Note: The black circle corresponds to the all‐meat control (0% extender). Error bars presented are the calculated standard deviations within each treatment. Data points with an *indicate a significant difference from the control (Dunnett, *p *=* *0.05)

Although meat extender type and meat extender concentration did not significantly affect lightness (L*) and red color (a*) (data not shown), both variables had a significant effect on yellow color (b*) (Table 3). Figure 3 shows changes in patty yellow color, b*, values as a function of meat extender type and concentration. The addition of mushrooms to beef patties at concentrations as low as 10% by weight significantly increased yellow color, b*, values when compared to the all‐meat control, while yellow color remained statistically similar at all levels of TSP concentration. The caramel color of the TSP, similar to that of cooked ground beef, may have contributed to the absence of differences in this parameter when compared to the all‐meat control. Previous research on oyster mushroom substitution into chicken patties showed that yellow color values decreased at concentrations of 25% and higher. Increased yellow (*b) values were also found in patties substituted with soy. The addition of 2% SPI to pork patties significantly increased yellow color values while an addition of 30% TSP to beef patties significantly decreased them (Akesowan, 2010; Deliza, Serna Saldivar, Germani, Benassi, & Carbral, 2002). Color results are greatly influenced by the color of constituent of the formulation (e.g., meat, mushroom, and soy type), which makes comparing results and generalizing a meat extender's effect on color difficult.

#### 3.1.4 Mechanical tests

**Figure 4:**
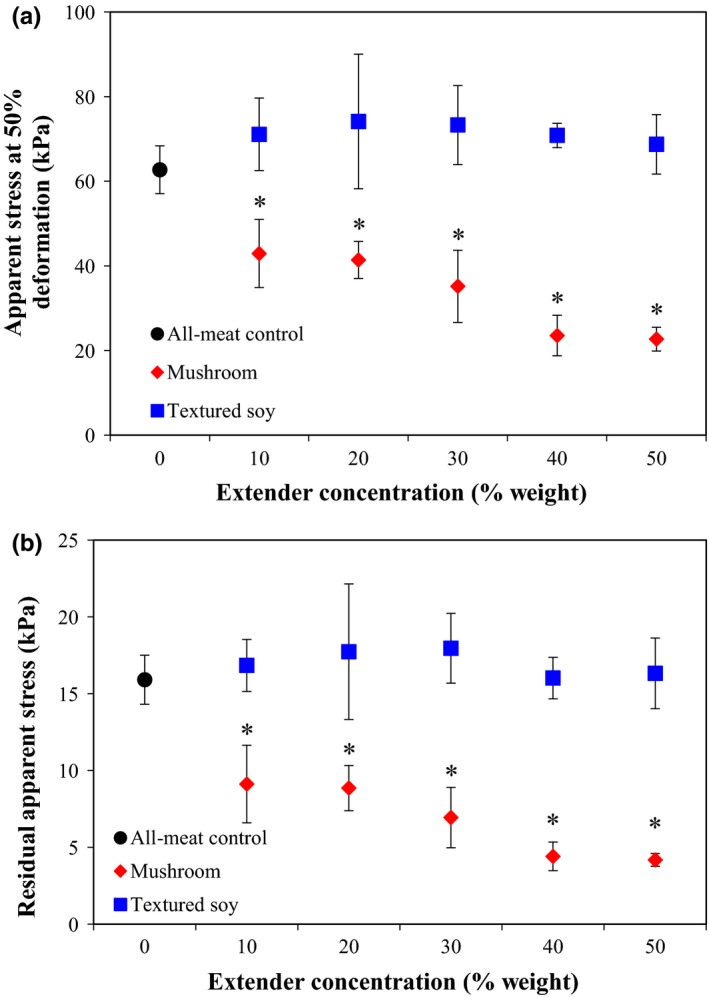
Effect of meat extender type (mushroom or textured soy) and concentration on (a) apparent stress at 50% deformation and (b) residual apparent stress. *Note*: The black circle corresponds to the all‐meat control (0% extender). Error bars presented are the calculated standard deviations within each treatment. Data points with an *indicate a significant difference from the control (Dunnett, *p *=* *0.05)

Meat extender type and concentration have significant effects on both apparent stress at 50% deformation and residual apparent stress (Table 3). Figures 4a and 4b showed that the addition of mushrooms at any concentration significantly reduced the patty's consistency when compared to the all‐meat control. Due to the heterogeneity of the meat extended formulations, there was a noticeable variation within each treatment; however, there were no statistically significant differences in either mechanical attribute toward the values obtained for the all‐meat control and TSP meat extender products across all concentrations. Wong et al. (2017) also identified mechanical differences in meat‐based products, specifically taco filling substituted with mushrooms. Apparent stress at 50% deformation significantly decreased at 50% substitution, while the residual apparent stress decreased at lower substitution levels, for example, 25%. Wan Rosli et al. (2011) reported lower values of selected textural characteristics for chicken patties substituted with 25% and 50% blanched oyster mushroom. The mushroom substituted samples displayed lower levels of hardness compared to the all‐meat control. Studies on the effect of the addition of TSP on mechanical properties and textural characteristics of patties do not show a clear trend. While beef and pork patties substituted with 3% SPI and 2–5% TSP displayed lower levels of hardness than their all‐meat controls (Akesowan, 2010 and Kassama, Ngadi, and Raghavan, 2003), pork patties substituted with 2–10% SPI showed increased hardness levels (Danowska‐Oziewicz, 2014). The lack of a discernible trend in mechanical and textural properties can be attributed to the large variability in soy‐based meat extenders options as soy ingredients are derive with different composition (e.g., soy type), processing conditions (cooking time and temperature), and to a lower extent to the methodology in which to assess the changes (compression–relaxation tests vs. Texture Profile Analysis). Based on the response from the mechanical and textural characteristic data, h‐TSP has a more meat‐like quality compared to mushroom meat extenders in a patty application. However, it should be noted that instrumental differences do not always correlate with sensory perceived differences (Corradini, Engel, & Peleg, 2001; Chanasattru, Corradini, & Peleg, 2002). Therefore, sensory testing is required to identify if these similarities or differences would be important in influencing acceptability from a consumer perspective.

#### 3.1.5 Sodium content

**Figure 5:**
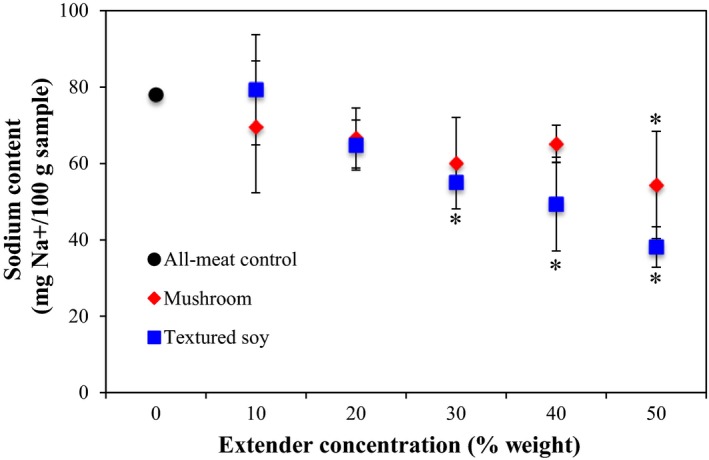
Effect of meat extender type (mushroom or textured soy) and concentration on sodium content. *Note*: The black circle corresponds to the all‐meat control (0% extender). Error bars presented are the calculated standard deviations within each treatment. Data points with an *indicate a significant difference from the control (Dunnett, *p *=* *0.05)

Mushrooms and h‐TSP have lower sodium contents than ground beef (5 mg/100 g, 3 mg/100 g, and 66 mg/100 g, respectively), which is reflected in the general reduction in sodium content of the beef patties with increasing meat extender concentration in Figure 5 (U.S. Department of Agriculture, 2016). Statistical analysis, with the Dunnett's Test, helped to identify the minimum concentration of each meat extender needed to significantly reduce sodium content. Figure 5 shows that mushroom substitution below 50% did not significantly reduce sodium content, while TSP substitution had an impact on sodium content at concentrations of 30% and above. This suggests that TSP can reduce sodium in beef patties when added at lower levels than mushroom even though it has higher sodium content than mushrooms (10 mg/100 g vs. 5 mg/100 g, respectively) due to dilution effect of hydrating the TSP prior to blending with into the beef patty. Although the research on sodium reducing strategies is extensive, the effect of the systemic addition of mushrooms and TSP on sodium reduction has historically not been reported.

#### 3.1.6 Fat content and retention

**Figure 6:**
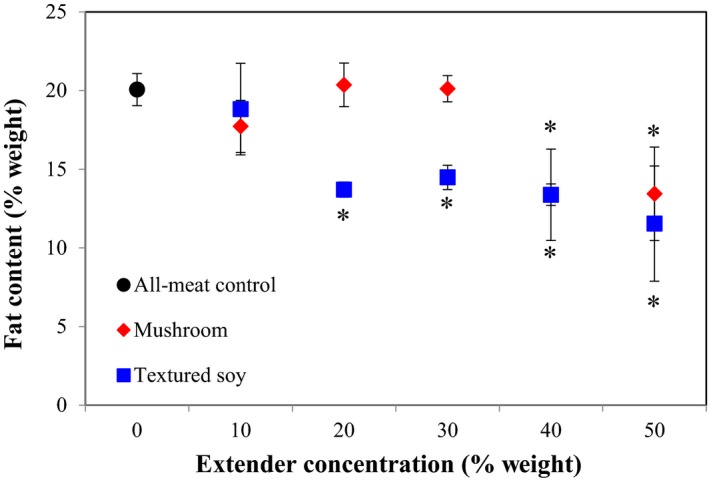
Effect of meat extender type and concentration on fat content. *Note*: The black circle corresponds to the all‐meat control (0% extender). Error bars presented are the calculated standard deviations within each treatment. Data points with an *indicate a significant difference from the control (Dunnett, *p *=* *0.05)

Mushrooms and TSP have lower fat content than 80/20 ground beef (0.34 g/100 g, 1.30 g/100 g, and 20 g/100 g before cooking, respectively), which was reflected in the general decrease in fat content with increased meat extender substitution in Figure 6 (U.S. Department of Agriculture, 2016). Statistical analysis, with the Dunnett's Test, was used to detect significant differences in fat content between the all‐meat control and variant formulations. Figure 6 shows that the addition of mushrooms levels of 40% and higher significantly reduced fat content, while TSP required only substitutions of 20% to have a significant effect on fat content. Wan Rosli & Solihah (2012, 2014) also showed that the addition of blanched oyster mushrooms to chicken and beef patties at concentrations of 25% and higher significantly reduced fat content. Pork patties supplemented with 2% to 10% SPI had significantly lower fat contents compared to its all‐meat counterpart (Danowska‐Oziewicz, 2014). Statistical analysis did not show any variation in fat retention among the all‐meat control and variant formulations regardless of meat extender type or concentration (Table 3).

Overall, monitoring changes in composition and physical properties due to the addition of meat extenders showed how mushroom and textured soy affected beef patties differently. In general, the addition of mushrooms to beef patties resulted in increased moisture and yellow color, decreased mechanical properties, sodium and fat content, and maintained a similar yield, lightness, and red color in comparison with the all‐meat control. The substitution of meat by h‐TSP resulted in increased yield, decreased sodium and fat content, and maintained similar moisture, color, and textural characteristics in comparison with the all‐meat control. Although the changes were dose dependent, there was not a clear cutoff value that could be deemed as the top performer. Therefore, concentrations of 10%, 20%, and 30% extender by weight were selected for further testing with sensory analysis to help identify an optimal concentration of meat extender that enhances the nutritional aspects of the beef patties while maintaining their typical physical properties.

### 3.2 Hedonic sensory study optimizing meat extender concentration

Based on composition and physical characteristics, h‐TSP substitutions would seem more advantageous than mushroom in beef patties due to the more statistical similarity to the all‐meat control. However, these similarities might not directly correspond with consumer acceptance. Therefore, sensory analysis was conducted to determine whether the physical differences detected by analytical methods would translate into a difference deemed important by consumers. An initial hedonic sensory test was performed to evaluate consumer liking of all‐meat and meat extender patties using the same formulations prepared for physical characterization. Beef patties were comprised of 80/20 ground beef, meat extender, and salt. The control formulation consisted of 98.5% meat, 0% meat extender, and 1.5% salt by weight. Variant formulations used 10%, 20%, or 30% IQF mushroom or h‐TSP by weight based on the samples that exhibited the most similar physical characteristics to the control. Meat blend and salt level were kept constant throughout the all‐meat control and variant formulations to solely evaluate the effects of meat extender concentration on consumer liking (Table 2).

The Hedonic Test #1 used 55 consumer panelists. Table 4 shows that the overall liking of variant formulations regardless of meat extender type or concentration was similar to the all‐meat control. Variant formulation aroma, flavor, juiciness, saltiness, and overall liking were also statistically similar to the all‐meat control. However, color and texture scores varied among the formulations with statistical difference demonstrated with asterisks (Table 4). Test subjects liked the color of the all‐meat formulation similar to all TSP and mushroom variant formulations; however, there were statistical differences in liking scores when comparing the usage ratios against the different extenders. The 10% mushroom variant was reported as statistically different from the 20% and 30% mushroom variants, respectively. This suggests that the differences in yellow color influenced by mushroom substitution (Figure 3) may also affect consumer liking. In addition, the two‐way ANOVA detected a significant difference in texture liking among the formulations, further analysis with Tukey's HSD Test showed that the texture of the all‐meat formulation was statistically similarly liked to five of the six variant formulations with the exception of the formulation containing 20% TSP (highest liking score). This finding suggests that even though mushroom substitution significantly decreases the patty's mechanical properties (Figure 4a and 4b), it does not influence consumer liking. The texture hedonic findings indicate that the use of up to 30% mushrooms is acceptable in textural quality for panelists while the results from the TSP indicate that there may be an optimal usage level (around 20%) before the quality declines.

**Table 4:** Average liking values of the sensory attributes for the burger patty hedonic study

Formulation	Overall	Aroma	Color**	Flavor	Juiciness	Saltiness	Texture*
100% Ground Beef (Control)	5.70 ± 1.72a	5.72 ± 1.63a	5.41 ± 1.91ab	6.15 ± 1.73a	5.35 ± 1.93a	5.59 ± 1.57a	4.98 ± 2.03b
10% Mushroom	6.19 ± 1.74a	6.08 ± 1.85a	6.27 ± 1.91a	6.27 ± 1.76a	6.27 ± 1.66a	5.69 ± 1.64a	5.58 ± 2.21ab
20% Mushroom	5.61 ± 1.81a	5.04 ± 2.20a	4.43 ± 2.12b	5.68 ± 1.79a	6.18 ± 1.68a	5.68 ± 2.09a	5.89 ± 2.02ab
30% Mushroom	5.08 ± 2.43a	5.54 ± 2.00a	4.27 ± 2.07b	5.62 ± 2.38a	6.15 ± 1.83a	5.23 ± 2.03a	5.81 ± 2.02ab
10% TSP	6.18 ± 1.93a	5.96 ± 1.45a	5.68 ± 2.02ab	6.25 ± 1.67a	6.00 ± 1.59a	6.00 ± 1.54a	6.04 ± 1.50ab
20% TSP	6.28 ± 1.69a	5.83 ± 1.07a	5.72 ± 2.23ab	6.10 ± 1.42a	6.14 ± 1.57a	5.97 ± 1.40a	6.55 ± 1.38a
30% TSP	5.24 ± 2.17a	5.28 ± 1.91a	5.21 ± 1.97ab	5.14 ± 2.20a	5.00 ± 2.15a	5.48 ± 1.57a	5.86 ± 1.88ab

*Note*: Samples with at least one similar letter label within each column are statistically similar based on two‐way ANOVA; Tukey's HSD test *p = 0.05; *^*^ *p *= 0.01.

Previous sensory research on patties extended with mushroom or soy products has resulted in a diverse array of findings, probably due to differences in meat extender products and testing procedures. For example, chicken and beef patties substituted with 25% and 50% blanched oyster mushroom scored statistically similar hedonic values as the all‐meat control in overall acceptability, color, flavor, texture, and juiciness with untrained panelists (Wan Rosli & Solihah, 2012, 2014). In contrast, the results from this work displayed statistical differences with color being less liked when the mushroom usage level as at 10% and 20% indicating that the mushroom type, in this case white mushrooms, may influence the consumer acceptance. Unformed beef applications, such as taco‐based fillings, have also shown acceptable sensory quality attributes using white button mushrooms up to levels as high as 45% (Wong et al., 2017).

The addition of up to 3% SPI in pork patties showed decreased liking of patty overall acceptability, color, flavor, and texture (Akesowan, 2010). Similarly, a trained panel scored pork patties with 10% SPI less acceptable than its all‐meat counterpart (Danowska‐Oziewicz, 2014). Substitution of 30% and 45% TSP into beef patties received lower liking scores for flavor and texture from trained panelists; however, overall acceptability remained statistically similar to the all‐meat control (Deliza et al., 2002; Rentfrow, Brewer, Weingartner, & McKeith, 2004). In our findings, the usage of up to 30% TSP did not exhibit any statistical differences compared to an all‐beef control. However, the type of TSP product used for our soy‐based meat extenders was a soy concentrate base (Protein 65%–90%), whereas the samples prepared in the other presented studies used a defatted soy flour‐based TSP (48%–55% Protein) which may have attributed to these organoleptic differences. Brewer, McKeith, and Britt (1992) have also observed lower liking scores of for beef patties that use soy flour‐based textured protein meat extenders compared to soy concentrates at the same ratio, which supports that different TSP types may influence the sensory attributes. Different meat, mushroom, and soy types, as well as the varied concentration of each patty ingredient may also influence the differences between current findings and previous research.

The results of this test indicated that consumers equally like the all‐meat control and a beef patty containing up to 30% mushroom or TSP, but may not like the color of the mushroom substituted patties to the same extent. Therefore, 20% substitution level was selected for further testing to evaluate whether this substitution could also be a feasible strategy for sodium reduction in patties. The selection of this substitution level allowed maximized extender usage in the beef patties while imparting minimal differentiation from the all‐meat control.

### 3.3 Hedonic sensory study of reduced sodium patties

A second hedonic sensory test was conducted to evaluate consumers’ acceptability of meat extenders in beef patties as a means to reduce sodium in a beef patty application. In the evaluated formulations, two salt levels and three beef formulations using both extenders were tested. Again, patties were comprised of 80/20 ground beef, meat extender, and salt with the control formulation consisting of 98.5% meat, 0% meat extender, and 1.5% salt by weight. The meat blend was kept constant throughout the all‐meat full sodium control (1.5% salt) and variant formulations to solely look at meat extenders’ potential mitigating effects of flavor loss in reduced sodium products (1.1%). All variant formulations used the “reduced sodium” level and the level of meat extender was set at 20% for both mushroom and h‐TSP (Table 2).

This hedonic sensory test used 56 untrained consumer panelists. Table 5 shows that overall liking of reduced sodium formulations containing 0% extender and 20% mushroom was similar to the all‐meat full sodium control while the reduced sodium formulation containing 20% TSP was liked statistically less. This could be attributed to the differences in formulation liking across the other test attributes as detected by the statistical analysis. The all‐meat formulations received similar liking scores for aroma, while the aroma of the extended formulations was not as favorable. The color of the reduced sodium 20% TSP formulation was similarly liked to the all‐meat full sodium control while the reduced sodium 20% mushroom formulation received significantly lower liking scores. Similar to the previous hedonic study, this suggests that the differences in patty yellow color due to mushroom substitution (Figure 3) may also influence consumer liking and that a color correction might boost the acceptability of the substituted product. Both reduced sodium meat extended formulations had less favorable flavor and aroma compared to the all‐meat full sodium control and the reduced sodium formulation with no extender. The juiciness of the reduced sodium 20% mushroom formulation was most liked, followed by the two all‐meat formulations, and the reduced sodium 20% TSP formulation was least liked. Probably, the significant increase in moisture content due to mushroom substitution (Figure 1a) might have a positive effect on juiciness and consumer liking. Finally, variant formulation saltiness and texture liking were statistically similar to the all‐meat full sodium control. The findings on texture were consistent with the previous hedonic study, which suggested that although mushroom substitution significantly decreased the patty's consistency (Figure 4a and 4b), it did not influence consumer liking. The results from this test showed that consumers might equally like the all‐meat full sodium control and a reduced sodium beef patty containing 0% or 20% mushroom, but again might not like the color of the mushroom substituted patties compared to the control.

**Table 5:** Average liking of the sensory attributes for the hedonic study on meat extension in reduced sodium burger patties

Formulation	Overall**	Aroma**	Color**	Flavor**	Juiciness**	Saltiness	Texture
100% Ground Beef, 1.5% Salt (Control)	5.95 ± 1.81a	5.98 ±1.79a	5.54 ±1.79b	6.43 ±1.82a	5.68 ±2.03b	5.77 ±2.05a	5.20 ±2.42a
100% Ground Beef, 1.1% Salt	6.17 ±1.53a	6.30 ±1.34a	6.24 ±1.71a	6.28 ±1.68ab	5.61 ±1.76b	5.80 ±1.62a	5.57 ±1.98a
20% Mushroom, 1.1% Salt	5.56 ±2.05ab	5.30 ±1.64b	4.41 ±2.14c	5.67 ±1.93bc	6.74 ±1.53a	5.83 ±1.86a	5.87 ±2.04a
20% Textured Soy, 1.1% Salt	5.05 ±1.90b	5.18 ±1.85b	4.98 ±1.80bc	4.98 ±2.01c	4.91 ±1.96c	5.32 ±1.86a	5.29 ±1.91a

*Note*: Values within a column with at least one similar letter label are statistically similar (ANOVA, *p = 0.05, **p = 0.01; Duncan's New Multiple Range Test, significant difference letter labels by column).

## 4 CONCLUSIONS

This work focused on determining the acceptability of mushroom‐based meat extension as a viable sodium reduction strategy in beef patty applications by comparing it to a traditional meat extender, TSP, at varying concentrations and to and all‐beef control. The physical properties of the samples suggested that increasing the level of mushroom extender would perform statistically similar to an all‐meat control in yield, lightness (L* value), and red color (a* value) while increasing the moisture and yellow color (b* value) and decreasing the mechanical properties, sodium content, and fat content of the final products. Increasing the concentration of TSP as a meat extender resulted in higher yield, lower sodium, and fat content and did not affect moisture, color, and texture again when compared to an all‐meat control. Hedonic sensory analysis showed that variations in meat extender type and concentration did not affect consumer overall liking, aroma, flavor, saltiness, and juiciness liking scores in full sodium beef patties. However, meat extension using mushrooms yielded liking scores more similar to the all‐meat formulations than TSP in reduced sodium applications. Reduced sodium patties containing mushroom received statistically similar overall liking scores to its all‐meat, full sodium counterpart while the reduced sodium patties containing TSP did not. The findings from this research suggest that mushrooms have the potential to be successfully incorporated into reduced sodium meat products to provide a healthier product.

## ETHICAL STATEMENT

This study was approved by the Institutional Review Board of the University of Massachusetts Amherst.
